# Differential placental expression profile of human *Growth Hormone*/*Chorionic Somatomammotropin* genes in pregnancies with pre-eclampsia and gestational diabetes mellitus

**DOI:** 10.1016/j.mce.2012.02.009

**Published:** 2012-05-15

**Authors:** Jaana Männik, Pille Vaas, Kristiina Rull, Pille Teesalu, Maris Laan

**Affiliations:** a Human Molecular Genetics Group, Institute of Molecular and Cell Biology, University of Tartu, Riia 23, 51010 Tartu, Estonia; b Women’s Clinic of Tartu University Hospital, L. Puusepa 8, 51014 Tartu, Estonia

**Keywords:** h*GH*/*CSH* genes, Placental expression, Pre-eclampsia, Gestational diabetes mellitus, Alternative mRNA transcripts, Fetal growth

## Abstract

The human *GH*/*CSH* cluster consisting of one pituitary-expressed (*GH1*) and four placenta-expressed loci has been implicated in maternal metabolic adaptation to pregnancy, regulation of intrauterine and postnatal growth. We investigated how the mRNA expression profile of placental *GH2*, *CSH1* and *CSH2* genes and their alternative transcripts correlates with maternal pre-eclampsia (PE) and/or gestational diabetes mellitus (GD). The expression of studied genes in PE placentas (*n* = 17) compared to controls (*n* = 17) exhibited a trend for reduced transcript levels. The alternative transcripts retaining intron 4, *GH2-2* and *CSH1-2* showed significantly reduced expression in PE cases without growth restriction (*P* = 0.007, *P* = 0.008, respectively). In maternal GD (*n* = 23), a tendency of differential expression was detected only for the *GH2* gene and in pregnancies with large-for-gestational-age newborns. Our results, together with those reported by others, are consistent with a pleiotropic effect of placental h*GH*/*CSH* genes at the maternal-fetal interface relating to the regulation of fetal growth and the risk of affected maternal metabolism.

## Highlights

► A trend for lower level of all placental h*GH*/*CSH* transcripts in preeclampsia. ► Reduced *GH2-2*/*CSH1-2* transcripts retaining intron 4 only in preeclampsia without SGA. ► Increased level of placental *GH2* mRNA in gestational diabetes with LGA newborns. ► h*GH*/*CSH* genes exhibit pleiotropic effects on fetal growth and maternal metabolism.

## 1 Introduction

Human placental growth hormone (PGH) and placental lactogen (PL) have been suggested to be crucial players in maternal metabolic adaptation to pregnancy as well as in fetal growth and development (Freemark, 2006; Handwerger, 2009; Handwerger and Freemark, 2000). These hormones are encoded by the human *Growth Hormone*/*Chorionic Somatomammotropin* (h*GH*/*CSH*) locus situated on chromosome 17q22-24 (George et al., 1981). The locus contains five duplicated genes that are highly similar at the DNA level (92–98%), but exhibit substantial heterogeneity in their diversity patterns (Sedman et al., 2008). Four of these genes – *GH2*, *CSH1*, *CSH2*, and *CSHL1* – are expressed in placenta, while a single ancestral *GH1* gene is expressed exclusively in the anterior pituitary (Barsh et al., 1983; Chen et al., 1989). *GH1* encodes pituitary growth hormone (GH), which is involved in the regulation of postnatal growth and adult metabolism. *GH2* encodes PGH, while the *CSH1* and *CSH2* genes encode identical mature protein PL, alternatively named also as chorionic somatomammotropin (CSH). *CSHL1* has been shown to be expressed in placenta (Hu et al., 1999; MacLeod et al., 1992; Misra-Press et al., 1994; Männik et al., 2010), although no protein has been reported.

The expression of four highly conserved and structurally similar h*GH*/*CSH* genes, *GH2*, *CSH1*, *CSH2*, *CSHL1*, is coordinately induced during fetal development in the syncytiotrophoblast layer of placenta, increasing between 12 and 20 weeks of gestation and then plateauing through term (MacLeod et al., 1992). Multiple alternative splicing products with differential expression of these genes can be observed in placental tissue (MacLeod et al., 1992; Männik et al., 2010). The main splicing pattern is shared by *GH1-1*, *GH2-1*, *CSH1-1* and *CSH2-1* transcripts, which are formed by joining of five canonical exons. These mRNAs code for mature hormones of 191 amino acids with 85% or greater peptide sequence identity (MacLeod et al., 1992). Three alternative mRNA splicing products have been described for the *GH2* (Boguszewski et al., 1998; Cooke et al., 1988a,b; Männik et al., 2010). The most prominent alternative splicing product of *GH2* (*GH2-2*) retain intron 4. The *GH2-3* transcript results from the usage of an alternative donor splice-site within exon 4 causing a 4 bp deletion. Both *GH2* alternative splice products contain a frameshift resulting in a putative protein with unique C-terminus. The minor *GH2-4* transcript skips the regular acceptor splice-site and uses an alternative site 45 bp downstream within the exon 3. A small percentage of *CSH* transcripts also retain intron 4 (*CSH1-2*, *CSH2-2*) through gestation, the majority derived from the *CSH1* gene (MacLeod et al., 1992; Männik et al., 2010). The profile of alternative splice-products of *CSHL1* differs from other h*GH*/*CSH* genes due to the loss of the traditional donor splice-site in intron 2 (Männik et al., 2010; Misra-Press et al., 1994). The majority of *CSHL1* mRNA pool lacks a signal peptide coding exon 2 (Männik et al., 2010) and therefore is unable to express secreted protein.

Both placental hormones, PGH (coded by *GH2-1* transcript) and PL (coded by *CSH1-1* and *CSH2-1* mRNA), are mainly secreted into the maternal blood stream and act by binding to somatotrophic and lactogenic receptors on a wide variety of tissues and have biological actions in many tissues (Handwerger, 2009). Their levels increase markedly in maternal circulation in progression of pregnancy. Lower than normal plasma concentrations of PL and PGH have been determined in women with fetal intrauterine growth restriction (IUGR)/small-for-gestational-age (SGA) pregnancies (Chellakooty et al., 2004; Mirlesse et al., 1993; Spellacy and Buhi, 1976). Consistently, we have demonstrated that the majority of pregnancies with a SGA newborn show down-regulation of the entire h*GH*/*CSH* cluster in placenta, whereas in the case of large-for-gestational-age (LGA) newborn, the placental expression of *CSH* major transcripts (*CSH1-1*, *CSH2-1*) was significantly increased compared with appropriate-for-gestational-age (AGA) newborns (Männik et al., 2010). In addition to the effect on fetal growth, aberrations in secretion of both hormones have been reported in common maternal pathological conditions of pregnancy like gestational diabetes mellitus (GD) and pre-eclampsia (PE) (reviewed in Handwerger, 2009). Although, there is abundance of studies on GD and PE, the data on the expression profiles and the use of alternative splicing pathways of hormone coding human placental *GH2*, *CSH1* and *CSH2* genes in these complications, is limited. Detailed studies of the h*GH*/*CSH* cluster have been hindered by high-sequence identity (>90%) among the genes and extensive usage of alternative splicing in multiple mRNA transcripts.

The present study aimed to examine the expression of alternative mRNA transcripts of *GH2*, *CSH1* and *CSH2* genes in placental samples in case of maternal pregnancy-related complications of GD and PE (symptoms > 34 week). We applied a sensitive semi-quantitative assay capable of distinguishing alternatively spliced transcripts of individual placental h*GH*/*CSH* genes and their relative expression levels (Männik et al., 2010). Altered mRNA expression profile of h*GH*/*CSH* genes was identified in placentas from pregnancies complicated with PE and GD in comparison to uncomplicated pregnancies. In the case of GD, the birth-weight of newborns was identified as an additional confounder affecting the expression pattern of the h*GH*/*CSH* genes in placenta. These results in context with the previously published data suggest that the placental h*GH*/*CSH* genes might exhibit dual effects on fetal growth as well as on maternal metabolism.

## 2 Materials and methods

### 2.1 Study subjects

The study was approved by the Ethics Review Committee of Human Research of the University of Tartu, Estonia (permissions No. 146/18, 27.02.2006; 150/33, 18.06.2006; 158/80, 26.03.2007). A written informed consent to participate in the study was obtained from every family.

All subjects were recruited at the Women’s Clinic of Tartu University Hospital (2006–2011) in the framework of REPROMETA (REPROgrammed fetal and/or maternal METAbolism) sample collection. The study sample consisted of 57 pregnancies divided into three groups: 17 healthy women with uncomplicated pregnancies (defined as control group), 17 pregnancies complicated with pre-eclampsia (PE, symptoms > 34 week) and 23 with gestational diabetes mellitus (GD) (Table 1). All pregnancies were singleton. Fifty-three women out of fifty-seven delivered at term (gestational week 37–41). Two women in the control group and two PE patients had preterm labor at gestational week 36 (+1 up to +3 days). All multiparous women in the control group had had uncomplicated pregnancies in the past. Two women in the PE group had developed pre-eclampsia also in the previous pregnancy, and one patient had previously suffered from gestational blood pressure increase. Four women in the GD group also developed gestational diabetes in the previous pregnancy.

Among PE patients, 14 had severe and three mild form of the disease. Severe form of PE was defined as hypertension (systolic blood pressure ⩾160 mm Hg and/or diastolic blood pressure ⩾110 mm Hg) and/or proteinuria of ⩾5 g in 24 h. Mild form of PE was diagnosed in case of increased systolic blood pressure ⩾140 mm Hg and/or diastolic blood pressure ⩾90 mm Hg) and/or proteinuria of ⩾0.3 in 24 h urine sample. According to the offspring’s birth-weight the PE patients were categorized as (i) PE with the birth of appropriate-for-gestational-age newborn (PE-AGA, *n* = 9) and (ii) PE with the birth of small-for-gestational-age newborn (PE-SGA, *n* = 8). Five of these SGA newborns were disproportionally small. SGA was diagnosed when the birth-weight of newborn was below the 10th percentile for the gender-adjusted gestational age in accordance to the growth curve estimated for Estonian population (Karro et al., 1997).

GD was diagnosed when 75 g oral glucose tolerance test (OGTT) performed at 24–28 weeks of gestation revealed either a fasting venous plasma glucose level of ⩾4.8 mmol/l, and/or at 1 h and 2 h plasma glucose level of ⩾10 mmol/l and ⩾8.7 mmol/l glucose, respectively. REPROMETA recruitment criteria had excluded patients with pre-gestational diabetes or diabetes with onset earlier than 20 weeks of gestation. The GD pregnancies were further classified according to the birth-weight of the newborn as AGA (GD-AGA, *n* = 12) or large-for-gestational age (GD-LGA, *n* = 11). Newborns were defined as LGA when the birth weight was greater than the 90th percentile for gender-adjusted gestational age (Karro et al., 1997). Two thirds of patients with GD (*n* = 9 (75%) in GD-AGA and *n* = 7 (64%) GD-LGA group) followed the low-glycemic diet and other got metformin.

All subjects were of white European ancestry and living in Estonia. Information regarding mother’s diseases, age, smoking, parity, parturition, childbirth history, and somatometric data was obtained from medical records during the course of pregnancy and after birth. Additionally, information regarding father’s diseases, age, height and weight was documented. Fetal outcome data collected at delivery included weeks of gestation, gender, birth-weight, birth length, head and abdominal circumference. Patients with multiple gestations, documented fetal anatomical anomalies or chromosomal abnormalities, diabetes mellitus, chronic hypertension and chronic renal diseases were excluded.

### 2.2 Tissue collection, RNA extraction and reverse transcription

Full thickness blocks of 2–3 cm were taken from the middle region of placenta within 2 h after cesarean section or vaginal delivery, placed immediately into RNAlater (Ambion Inc, Austin, TX, USA) and kept at −20 °C until RNA isolation. All samples have been collected by the same medical personnel.

Total RNA from 200 to 230 mg of tissue was homogenized and extracted using TRIzol® reagent (Invitrogen Life Technologies, Carlsbad, CA, USA) according to the manufacturer’s instructions. Isolated total RNA was purified using NucleoSpin® II Isolation Kit (Macherey–Nagel, Germany) and subsequently quantified by NanoDrop1000 Spectrophotometer (Thermo Scientific, USA). One microgram of total RNA was reverse transcribed to cDNA using Superscript III Reverse Transcriptase (Invitrogen Life Technologies) in accordance with manufacturer’s instructions.

### 2.3 Assay design and capillary electrophoresis

The assay design principles and the primer sequences have been described in detail previously (Männik et al., 2010). In brief, in order to discriminate between all described alternative transcripts of *GH2* and *CSH1*/*CSH2* genes the design of gene-specific RT-PCR primers had taken into account splicing pattern, polymorphic positions and tracks with high DNA sequence homology within the h*GH*/*CSH* gene cluster. The RT-PCR primer pair specific to the *GH2* gene distinguishes all four described transcripts by size (*GH2-1*: 364 bp, *GH2-2*: 619 bp, *GH2-3*: 360 bp, *GH2-4*: 320 bp). Additional reverse primer was designed into intron 4 of *GH2* gene to amplify specifically *GH2-2* transcript with comparable size (390 bp) to the other amplicons in order to facilitate simultaneous fragment analysis. Transcript-specific DNA fragments of *CSH* genes were obtained by parallel amplifications of the mixture of major products (*CSH1-1* and *CSH2-1*) and the pool of minor transcripts (*CSH1-2* and *CSH2-2*). RT-PCR was followed by gene-specific restriction digestion to distinguish between (i) *CSH1-1* and *CSH2–1* (EcoNI, Fermentas, Lithuania; restriction products 304 and 309 bp) or (ii) *CSH1-2* and *CSH2-2* (BstEII, Fermentas; fragments 358 and 401 bp). The RT-PCR conditions are given in the Supplemental data.

Equal amounts of the reference gene *GAPDH* combined with the gene-specific amplification product (*GH2*), or alternatively with the pool of DNA fragments from the restriction cutting of a PCR amplicon (*CSH* genes) were combined with Hi-Di™-Formamide (Applied Biosystems Inc., USA) including 0.2 μl internal size standard (MegaBACE ET400-R Size Standard, GE Healthcare, USA), and resolved on an Applied Biosystems 3130XL Genetic Analyzer.

### 2.4 Data analysis

All samples were amplified at least in triplicate. The relative expression level of each gene was calculated as follows: the value of the peak area (in relative fluorescent units) of each target gene was divided by the value of peak area of the reference gene (*GAPDH*) from the same lane of the electrophoresis. Intra-assay coefficient of variation was 10–15%.

The statistical analyses were performed using the statistical package SPSS version 17.0 (SPSS Inc., USA) and R 2.9.0, a free software environment for statistical computing and graphics (http://www.r-project.org/). Descriptive statistics are given as median values and ranges. Distributions of continuous phenotypic variables of the control group in comparison with patients groups (PE, GD) were tested by non-parametric Wilcoxon rank-sum test. Differences in relative gene expression between groups were assessed by Mann–Whitney *U* test and analysis of covariance (ANCOVA) using Bonferroni correction. No study-wide corrections for multiple tests were applied. Natural log-transformation was applied to all quantitative data to improve the approximation of normal distribution. The analysis comparing gene expression in PE and control placentas was adjusted for the birth-weight, birth length and abdominal circumference of the newborn, since these parameters differed between the two groups. After grouping PE cases according to the newborn’s birth-weight (PE-AGA, PE-SGA), the sub-groups differed significantly in gestational-age and this parameter was used as covariate in statistical tests for differential expression. Newborn’s birth-weight, birth length, head and abdominal circumference, placental weight as well as mother’s pre-pregnancy body mass index (BMI) were defined as confounders in the comparison of the maternal GD with the control group, and were used as covariates in testing differential expression of h*GH*/*CSH* genes. When GD pregnancies were grouped additionally by the newborn birth-weight (GD-AGA, GD-LGA), only mother’s pre-pregnancy BMI and age were used as covariates. *P* ⩽ 0.05 was considered as significant and *P* < 0.1 was considered suggestive.

## 3 Results

### 3.1 Characteristics of the study group

The study sample consisted of 57 singleton pregnancies – 17 uncomplicated (control group), 17 complicated with PE and 23 with GD diagnosis (Table 1). Women with uncomplicated and complicated pregnancies were similar in age, height, gestational weight gain, parity, smoking status, and gestational age. However, women with pregnancies complicated with GD had significantly higher pre-pregnancy BMI, heavier and taller newborns and placentas than the cases of uncomplicated pregnancies (*P* < 0.05). Newborns in PE group had significantly lower birth-weight compared to control group (*P* < 0.05). To avoid these confounding effects, comparisons of placental expression pattern of h*GH*/*CSH* genes transcripts between control and patients (PE, GD) group were adjusted for covariates as described in Materials and methods.

Gender effect on gene expression was addressed in the control sample by comparing the sub-groups stratified according to the sex of the newborn. h*GH*/*CSH* gene expression did not differ statistically (*P* > 0.1) in placentas obtained from the pregnancies resulting in the birth of female compared to male newborns (Suppl. Fig. 1).

### 3.2 Reduced expression of the entire h*GH*/*CSH* gene cluster in pre-eclamptic placentas

**Fig. 1:**
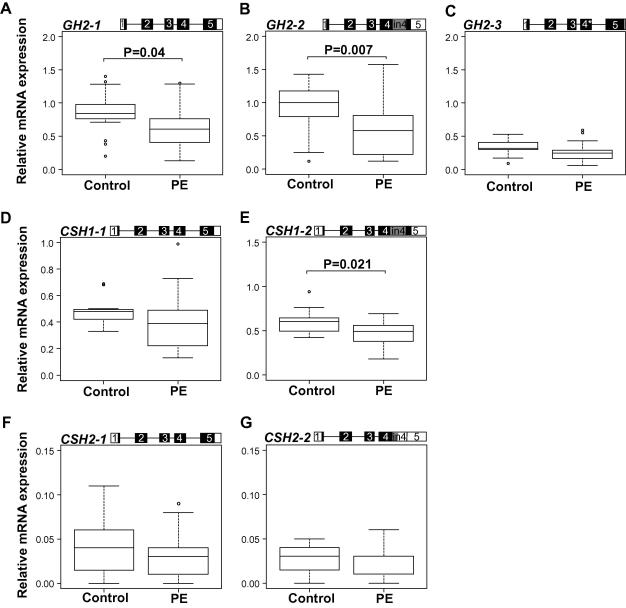
Comparison of relative expression levels of major alternatively spliced mRNA transcripts of *GH2* (A–C), *CSH1* (D and E), and *CSH2* (F and G) in placentas from uncomplicated pregnancies (control, *n* = 17) and complicated with pre-eclampsia (PE, *n* = 17). The corresponding transcript representing the presented data is shown on the top of each panel. Exons are shown as boxes and introns as lines. The coding region is denoted by black/grey-filled area. An asterisk (∗) denotes the 4 bp deletion in exon 4 of *GH2-3*. Relative expression of each transcript is given as ratio to the reference gene *GAPDH*. The boxes represent the 25th and 75th percentiles. The median is denoted as the line that bisects the boxes. The whiskers are lines extending from each end of the box covering the extent of the data on 1.5× interquartile range. Circles represent the outlier values. Plotted values are presented without covariate adjustment. Statistical differences between study groups were assessed by ANCOVA using Bonferroni correction. Statistical tests were adjusted for newborns birth-weight, birth length, and abdominal circumference. Bonferroni corrected *P*-values reflecting significant differences (*P* < 0.05) are shown.

In the pregnancies complicated with PE the placental expression profile of the entire h*GH*/*CSH* gene cluster showed a trend of reduced expression compared to the controls (Fig. 1A–G). The detected differential expression of PGH coding *GH2-1* mRNA transcript (*P* = 0.04) as well as the intron 4 retaining alternative mRNA splice variants *GH2-2* (*P* = 0.007) and *CSH1–2* (*P* = 0.021) was statistically significant (Fig. 1A, B, and E and Table 2). The two transcripts that code jointly for PL (*CSH1-1*, *CSH2-1*) showed a consistent trend towards lower gene expression, but the difference did not reach statistical significance (Table 2). Compared to placentas from uncomplicated pregnancies, the samples from the PE cases had an average 1.4- and 1.6-fold lower median expression levels for *GH2-1*, and *GH2-2*, respectively (Fig. 1A and B)*.* Level of *CSH* transcripts was reduced about 1.3-fold (Fig. 1E–G).

### 3.3 Low expression of *GH2-2* and *CSH1-2* in pre-eclampsia with unaffected fetal growth

**Fig. 2:**
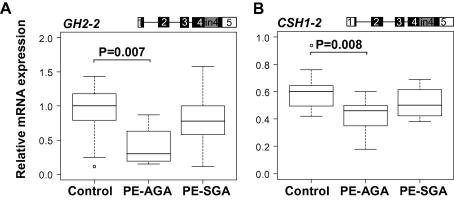
Differential expression of *GH2-2* (A) and *CSH1-2* (B), two alternative mRNA splice-variants retaining intron 4 sequence, in placental samples from pregnancies with pre-eclampsia (PE) grouped according to the birth-weight of the newborns – PE with the birth of appropriate-for-gestational age newborn (PE-AGA, *n* = 9) and PE with the birth of small-for-gestational age newborn (PE-SGA, *n* = 8), compared to control samples (*n* = 17). The corresponding transcript representing the presented data is shown on the top of panels. Exons are shown as boxes and introns as lines. The coding region is denoted by black/grey-filled area. Relative expression of each transcript is given as ratio to the reference gene *GAPDH*. The boxes represent the 25th and 75th percentiles. The median is denoted as the line that bisects the boxes. The whiskers are lines extending from each end of the box covering the extent of the data on 1.5× interquartile range. Circles represent the outlier values. Plotted values are presented without covariate adjustment. Statistical differences between study groups were assessed by ANCOVA using Bonferroni correction. Statistical tests were adjusted for gestational age. Bonferroni corrected *P*-values reflecting significant differences (*P* < 0.05) are shown.

PE can manifest itself as a maternal disorder without affecting fetal growth (newborns appropriate-for-gestational-age, PE-AGA) or it can occur in combination with a fetal growth restriction (PE-SGA; Table 1) (Grill et al., 2009; Huppertz, 2011). No differences were detected between the two subgroups of PE in the gene expression of PGH (*GH2-1*) and PL coding transcripts (*CSH1-1*, *CSH2-1*) (Suppl. Fig. 2). However, in the PE-AGA placentas the differential expression of the intron 4 retaining alternative mRNA splice variants *GH2-2* (*P* = 0.008) and *CSH1-2* (*P* = 0.010) (Fig. 2 and Table 2) was more pronounced than in the PE-SGA group. Compared to normal pregnancy, in the PE-AGA group the average expression of *GH2-2* and *CSH1-2* was approximately 2.2-fold (*P* = 0.007) and 1.4-fold down-regulated (*P* = 0.008) (Fig. 2).

### 3.4 Profile of h*GH*/*CSH* gene expression in placentas from gestational diabetes

**Fig. 3:**
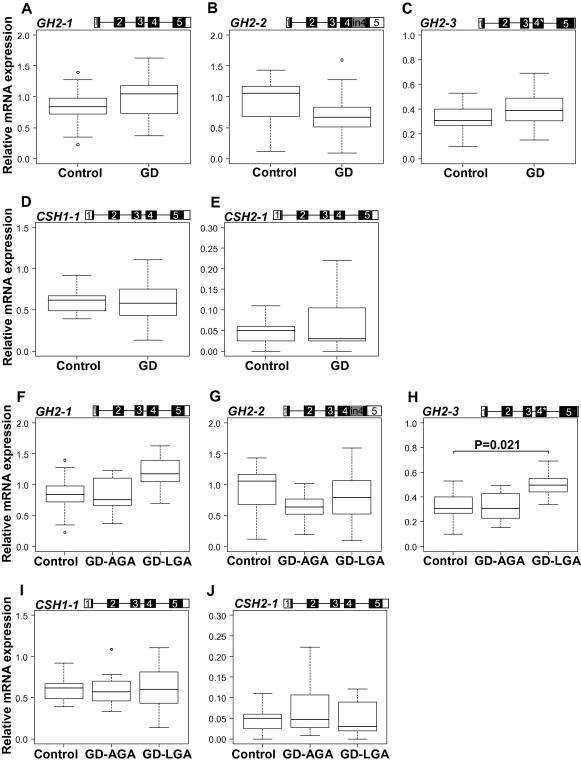
Comparison of relative expression levels of major alternatively spliced mRNA transcripts of *GH2*, *CSH1*, and *CSH2* in placentas from uncomplicated pregnancies (control, *n* = 17) and complicated with gestational diabetes mellitus (GD, *n* = 23) (A-E), as well as with GD pregnancies grouped by the birth-weight of newborns – GD associated with the birth of appropriate-for-gestational age newborn (GD-AGA, *n* = 12) and GD with birth of large-for-gestational age newborn (GD-LGA, n = 11) (F-J). The corresponding transcript representing the presented data is shown on the top of panel. An asterisk (∗) denotes the 4 bp deletion in exon 4 of *GH2-3*. Relative expression of each transcript is given as ratio to the reference gene *GAPDH*. The boxes represent the 25th and 75th percentiles. The median is denoted as the line that bisects the boxes. The whiskers are lines extending from each end of the box covering the extent of the data on 1.5× interquartile range. Circles represent the outlier values. Plotted values are presented without covariate adjustment. Statistical differences between study groups were assessed by ANCOVA using Bonferroni correction. Statistical tests were adjusted for newborn birth-weight, birth length, head and abdominal circumference, placental weight as well as mother’s pre-pregnancy BMI when comparing the control and GD group and for mother’s pre-pregnancy BMI and age after grouping GD samples by the newborn birth-weight. Bonferroni corrected *P*-values reflecting significant differences (*P* < 0.05) are shown.

In contrast to the PE group, no uniform trend for up- or down-regulation in placental expression of h*GH*/*CSH* cluster genes was detected in the cases of GD compared to the control group (Fig. 3A–E; Table 2). A trend towards higher median gene expression level of *GH2-1* and *GH2-3*, but a lower median expression of *GH2-2* mRNA transcripts was observed (Fig. 3A–C and Table 2), although statistically non-significant. No expressional alternations of placental *CSH* genes were detected in the analysis of the full study group of the maternal gestational diabetes (Fig. 3D and E and Table 2). GD can exhibit itself as a maternal disorder with the birth of an appropriate-for-gestational-age newborn (GD-AGA), or it can be accompanied by an accelerated fetal growth resulting in the birth of LGA newborn (GD-LGA) (Table 1). When the patients were grouped according to the offspring’s birth-weight, the detected increased expression of *GH2-1* (1.4-fold increase; *P* > 0.05; without Bonferroni correction *P* = 0.048) and *GH2-3* (1.5-fold increase; *P* = 0.021) transcripts was only characteristic to the GD-LGA placentas (Fig. 3F–H and Table 2). No expressional alternation of placental *CSH* genes was detected in the analysis of GD group divided by the newborns birth weight (GD-AGA, GD-LGA) (Fig. 3I–J and Table 2).

## 4 Discussion

This is the first study to systematically quantitate the placental gene expression profiles of *GH2*, *CSH1* and *CSH2* alternative mRNA transcripts in cases of maternal complications pre-eclampsia (PE, symptoms > 34 week) and gestational diabetes mellitus (GD) in comparison with uncomplicated pregnancies. The placentas from PE showed trend of reduced expression of the entire h*GH*/*CSH* gene cluster. Interestingly, a trend for reduced expression of all h*GH*/*CSH* genes in the cluster has been also described in the placentas from the pregnancies resulting in the birth of small-for-gestational-age (SGA) babies (Männik et al., 2010). Although, the pathophysiologies of PE and fetal growth restriction differ, alterations in the same pathways (e.g. inadequte spiral artery remodelation, syncytiotrophoblasts differentiation) might be involved (James et al., 2010). It has been also suggested that IUGR/SGA and PE-IUGR/SGA are two distinct pathologies each of which has a unique impact on trophoblast function (Newhouse et al., 2007; Huppertz, 2011). This may explain the observed differences in gene expression patterns of the placental h*GH*/*CSH* genes between the cases of PE-SGA reported in the current study, and uncomplicated pregnancies resulting in the birth of a SGA newborn described previously (Männik et al., 2010). In accordance with our study, in pregnancies with impaired blood flow, PE and IUGR, reduced serum levels of PGH (coded by *GH2-1*) have been measured (Schiessl et al., 2007). However, Mittal and co-workers have demonstrated increased median concentrations of PGH in both maternal and fetal circulations in case of PE compared to normal pregnancy (Mittal et al., 2007). This inconsistency may be explained by the selection criteria of the study group since the pathophysiology of PE is heterogeneous in origin as it is in presentation (Redman and Sargent, 2010).

As a novel finding, significant decrease of placental expression of *GH2-2* and *CSH1-2* appeared to be specific to the PE cases not accompanied by the growth restriction of the newborn. The *GH2-2* and *CSH1-2* are the most prominent alternatively spliced variants of *GH2* and *CSH1* genes forming about 25% and 7% of respective gene mRNA pool in term placenta (Männik et al., 2010). Both alternative transcripts retain intron 4 sequence and therefore encode new putative protein with different C-terminus (Cooke et al., 1988a; MacLeod et al., 1992). In contrast to PGH and PL coded by the major transcripts of *GH2*, *CSH1* and *CSH2*, the C-terminus of the putative proteins has a hydrophobic region typical for the membrane-located proteins (Cooke et al., 1988a; MacLeod et al., 1992; Untergasser et al., 2000). Both mRNAs are expressed until parturition with steadily increasing levels in the small fraction of syncytiotrophoblasts in placenta (MacLeod et al., 1992). Its possible location at the plasma membrane may imply to the function in cell-to-cell communication at the maternal-fetal interface. Alternatively, Untergasser and co-workers (2000) have proposed that CSH1-2 might function at the surface of the cell membrane by interacting with GH/PRL/cytokine receptors. The GH2-2 may exert a similar action. The exact role of the products of *GH2-2* and *CSH1-2* at the maternal-fetal interface has to be elucidated.

In both conditions of affected fetal growth, SGA and LGA, as well as in maternal PE the expression of the entire h*GH*/*CSH* gene cluster showed the uniform trend of either higher (LGA) or lower (SGA, PE) placental transcript levels. Maternal GD pregnancies contrasted this pattern. First, a trend for differential expression was only detected for the *GH2* gene. Secondly, the three *GH2* transcripts were not uniformly up- or down-regulated indicating to the possible role of altered alternative splicing shaping the gene expression profile. Thirdly, only the GD pregnancies accompanied with the birth of a large baby showed altered *GH2* gene expression patterns compared to the control group. In literature, a positive correlation has been confirmed between maternal serum PGH concentration and birth-weight in uncomplicated as well as diabetic pregnancies (Chellakooty et al., 2002; Fuglsang et al., 2003; McIntyre et al., 2000). In addition, it has been shown that transgenic mice over-expressing the gene encoding for PGH became larger than their normal littermates and developed hyperinsulinaemia and insulin resistance (Barbour et al., 2002). PGH has been suggested to act as potent insulin antagonist that stimulates maternal lipolysis and thereby promotes availability of sparse glucose and other nutrients for transplacental delivery and fetal growth (Newbern and Freemark, 2011).

Alterations in transcript profile of h*GH*/*CSH* genes in case of PE and GD can be explained by several mechanisms. In PE, reduced expression of the entire gene cluster might be mediated by polymorphisms or epimutations in locus control region (LCR) responsible for the functional activation of all h*GH*/*CSH* genes in tissue specific manner (Ho et al., 2004; Kimura et al., 2007). The expression of individual h*GH*/*CSH* genes might be further shaped by the allelic composition of the promoter or other regulatory sequence elements targeted by autocrine, paracrine or transcription factors. In GD, the *GH2* transcripts profile indicated to the possible role of shifted efficacy in usage of alternative splice-sites. This can be facilitated by mutations at splice sites or surrounding sequences, excess availability of certain splicing factor(s) or local chromatine modifications (Caceres and Kornblihtt, 2002; Cooper et al., 2009; Luco et al., 2010).

In summary, the placental gene expression profile of the entire h*GH*/*CSH* cluster in PE cases compared to the control group showed trend for reduced transcript levels and resembled the transcript profile described in placentas from the pregnancies resulting in SGA newborns (Männik et al., 2010). At the current stage it is not possible to differentiate whether reduced h*GH*/*CSH* gene expression is either causative or the consequence of these fetal and/or maternal complications. In contrast, the expression profile of h*GH*/*CSH* genes in maternal GD appeared to be altered only in cases resulting in the birth of a large baby. The conducted studies together with those reported by others indicated that the placenta-expressed h*GH*/*CSH* genes play an important dual role at the maternal-fetal interphase contributing to the regulation of fetal growth and in modulating the risk of affected maternal metabolism during pregnancy.

## Disclosure statement

None declared.

## Figures and Tables

**Table 1:** Parental and offspring characteristics of the family trios in the study.

	Study group		
	Control (*n* = 17)	PE (*n* = 17)	GDM (*n* = 23)
Mother:			
Age (year)	27 (20; 38)	28 (19; 39)	31 (21; 39)
Height (cm)	167 (160; 176)	168 (156; 178)	164 (150; 176)
BMI before pregnancy (kg/m^2^)	22 (19; 33)	24 (17; 33.5)	27 (18; 43)^a^
Gestational weight gain (kg)	14 (7; 31)	15 (6; 21.5)	14 (3; 27)
No. of women with nulliparity	10	11	8
No. of women smoking during pregnancy	0	0	1

Father:			
Age (year)	32.5 (21; 54)	30 (21; 46)	34 (22; 43)^b^
Height (cm)	181 (172; 187)	185 (175; 191)	181 (171; 198)^b^
BMI (kg/m^2^)	26 (20; 33)	24 (18.5; 38)	28 (21; 35)^b^

Offspring:			
Gestational age at birth (d)	275(254; 287)	265 (253; 288)	275 (253; 293)
Birth-weight (g)	3450 (2553; 4034)	2760 (1570; 4250)^a^	4046 (3154; 5420)^a^
Birth length (cm)	51 (45; 52)	48 (42; 51)^a^	52 (48; 55)^a^
Head circumference (cm)	34.5 (31; 37)	33.5 (29; 37)	36 (34; 38)^a^
Abdominal circumference (cm)	34 (30; 37)	32 (25; 37.5)^a^	36 (32; 40.5)^a^
IUGR	0	8	0
Macrosomia	0	0	11
Placental weight	510 (390; 790)	450 (320; 770)	659 (410; 1060)^a^
No. of boys/girls	9/8	12/5	10/13

Data are given as medians with ranges, except where indicated differently. PE, pre-eclampsia; GD, gestational diabetes mellitus; SGA, small-for-gestational-age; LGA, large-for-gestational-age.

a *P* < 0.05 vs. control group, Wilcoxon rank-sum test.

b Data of 18 fathers.

**Table 2:** Summary of adjusted and unadjusted statistical analyses for the placental h*GH*/*CSH* mRNA transcripts.

Controls vs. PE			Controls vs. PE cases grouped by birth weight: PE-AGA, PE-SGA				
mRNA transcript	MWU test	ANCOVA^∗^	MWU test		ANCOVA^∗^		
	Unadjusted *P*-value	Adjusted *P*-value	Unadjusted *P*-value		Adjusted *P*-value	Adjusted *P*-value	
			PE-AGA	PE-SGA		PE-AGA	PE-SGA
*GH2-1*	**0.028**	*F*(1, 27) = 4.634; ***P* = 0.040**	0.161	**0.026**	*F*(2, 29) = 2.288; *P* = 0.119	0.498	0.175
*GH2-2*	**0.015**	*F*(1, 27) = 8.416; ***P* = 0.007**	**0.004**	0.374	*F*(2, 29) = 5.658; ***P* = 0.008**	**0.007**	1.000
*GH2-3*	**0.047**	*F*(1, 27) = 2.028; *P* = 0.166	**0.094**	0.127	*F*(2, 29) = 0.957; *P* = 0.396	0.737	0.897
*CSH1-1*	0.061	*F*(1, 26) = 1.516; *P* = 0.229	**0.045**	0.300	*F*(2, 28) = 1.659; *P* = 0.208	0.259	0.970
*CSH2-1*	0.322	*F*(1, 26) = 0.051; *P* = 0.823	0.241	0.673	*F*(2, 28) = 0.739; *P* = 0.487	0.727	1.000
*CSH1-2*	**0.018**	*F*(1, 26) = 6.084; ***P* = 0.021**	**0.009**	0.219	*F*(2, 28) = 5.503; ***P* = 0.010**	**0.008**	0.337
*CSH2-2*	0.095	*F*(1, 26) = 0.942; *P* = 0.341	**0.017**	0.768	*F*(2, 28) = 0.946; *P* = 0.400	0.540	1.000

Controls vs. GD			Controls vs. GD cases grouped by birth weight: GD-AGA, GD-LGA				

mRNA transcript	MWU test	ANCOVA^∗^	MWU test		ANCOVA^∗^		
	Unadjusted *P*-value	Adjusted *P*-value	Unadjusted *P*-value		Adjusted P-value	Adjusted *P*-value	

GD-AGA	GD-LGA	GD-AGA	GD-LGA

*GH2-1*	0.288	*F*(1, 30) = 0.384; *P* = 0.540	0.674	**0.046**	*F*(2, 33) = 3.126; *P* = 0.057	1.000	0.143
*GH2-2*	0.071	*F*(1, 31) = 0.470; *P* = 0.498	**0.030**	0.424	*F*(2, 34) = 1.582; *P* = 0.220	1.000	0.518
*GH2-3*	0.074	*F*(1, 30) = 0.938; *P* = 0.341	0.982	**0.001**	*F*(2, 33) = 5.589; ***P* = 0.008**	1.000	**0.021**
*CSH1-1*	0.954	*F*(1, 30) = 0.201; *P* = 0.657	0.907	0.824	*F*(2, 33) = 0.208; *P* = 0.814	1.000	1.000
*CSH2-1*	0.798	*F*(1, 29) = 1.900; *P* = 0.179	0.555	0.855	*F*(2, 32) = 0.674; *P* = 0.517	0.766	1.000
*CSH1-2*	0.354	*F*(1, 29) = 0.360; *P* = 0.553	0.751	0.203	*F*(2, 32) = 1.455; *P* = 0.249	1.000	0.443
*CSH2-2*	0.976	*F*(1, 29) = 0.129; *P* = 0.723	0.902	0.937	*F*(2, 32) = 0.113; *P* = 0.894	1.000	1.000

MWU test, Mann–Whitney *U* test; ANCOVA, analysis of covariance; PE-AGA and PE-SGA, pre-eclampsia with the birth of appropriate-for-gestational-age or small-for-gestational-age newborn, respectively; GD-AGA and GD-LGA, gestational diabetes with the birth of AGA or large-for-gestational-age newborn, respectively. For ANCOVA the F-ratio, the degrees of freedom from which it was calculated and *P*-value are given. Additionally, *P*-values for Bonferroni corrected *Post hoc* pairwise comparisons between controls and cases grouped by the newborns birth weight are shown. Results with *P*-values ⩽ 0.05 are given bold. ^∗^Applied co-founder effects are described in Section 2.
