# Supplementary figures and images for: Differential placental expression profile of human Growth Hormone/Chorionic Somatomammotropin genes in pregnancies with pre-eclampsia and gestational diabetes mellitus
Source: Mol Cell Endocrinol. 2012 May 15;355(1):180–7. doi: 10.1016/j.mce.2012.02.009 (PMC3325480; doi:10.1016/j.mce.2012.02.009)

# Supplemental figure 1.

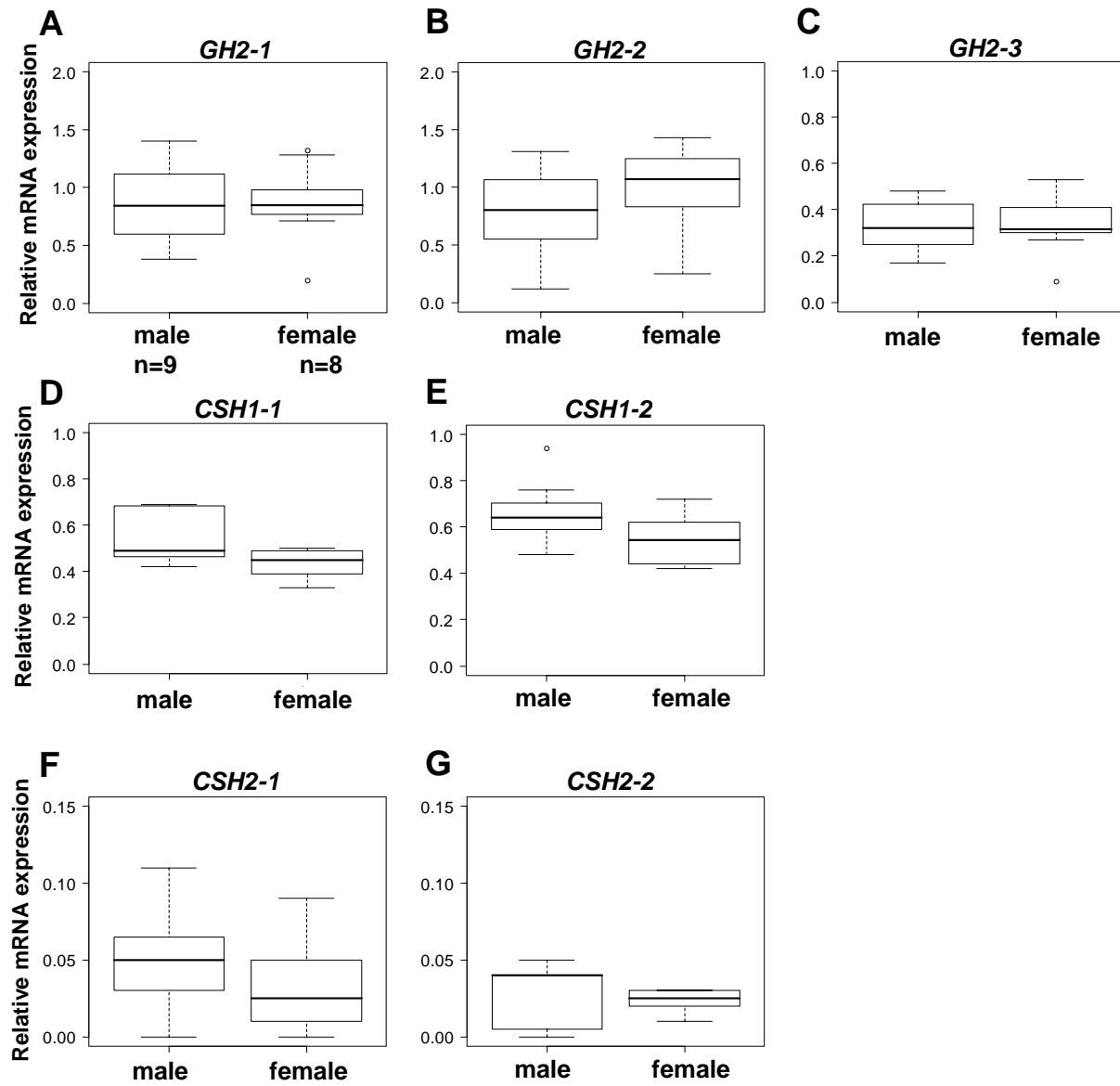

## Supplemental figure 2.

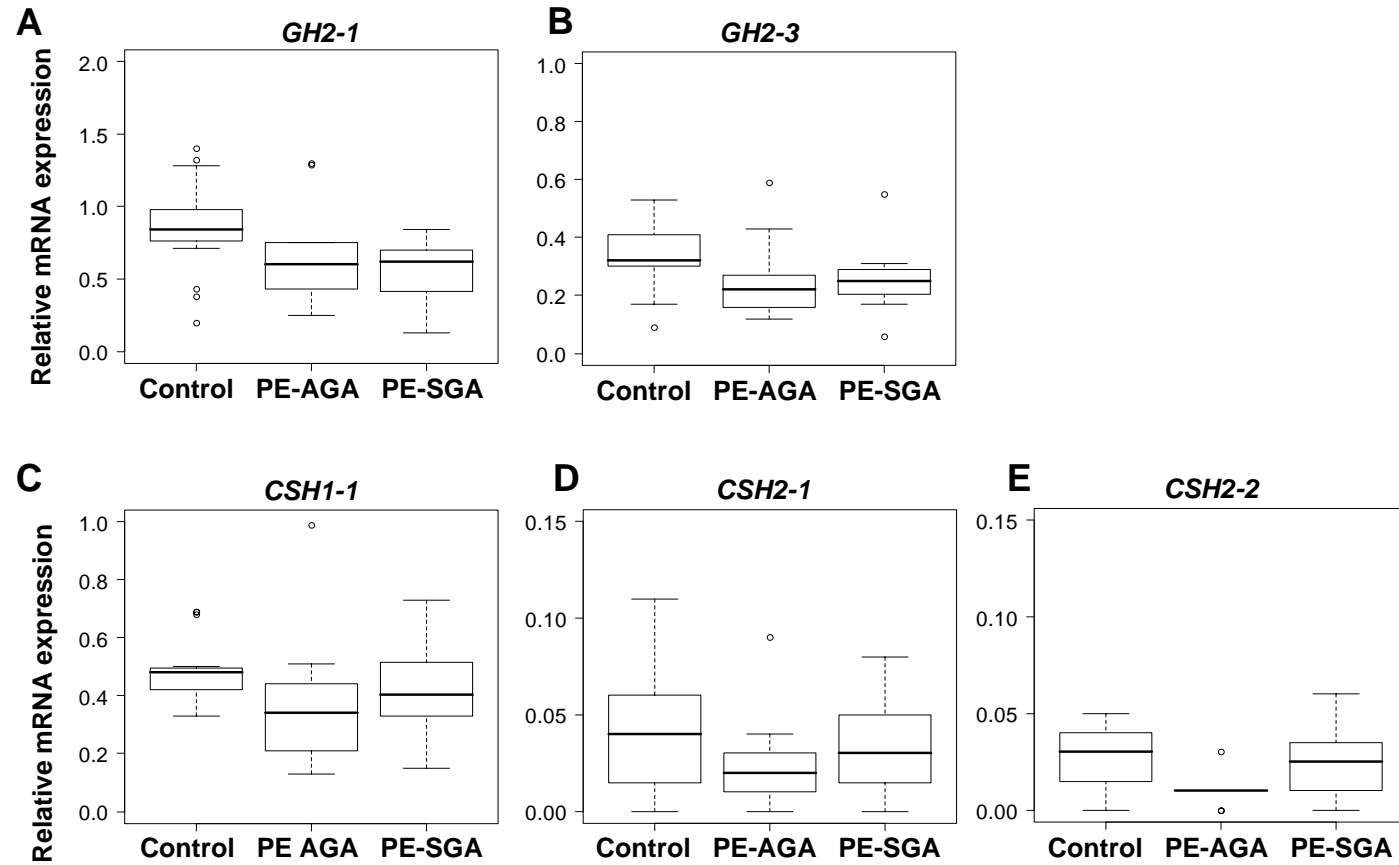

Supplement: Supplementary data 1 [file mmc1.pdf]
