# Supplementary material for: Differential placental expression profile of human Growth Hormone/Chorionic Somatomammotropin genes in pregnancies with pre-eclampsia and gestational diabetes mellitus
Source: Mol Cell Endocrinol. 2012 May 15;355(1):180–7. doi: 10.1016/j.mce.2012.02.009 (PMC3325480; doi:10.1016/j.mce.2012.02.009)
Supplement: Supplementary data 2 [file mmc2.doc]

**Title**: Differential placental expression profile of human *Growth Hormone/Chorionic Somatomammotropin* genes in pregnancies with pre-eclampsia and gestational diabetes mellitus

**Short title**: *GH/CSH* genes in placenta in case of PE and GD

**Key terms:** *GH/CSH* genes; placental expression; pre-eclampsia; gestational diabetes mellitus; alternative mRNA transcripts; fetal growth

**Authors:** Jaana Männik^1^, Pille Vaas^1,2^, Pille Teesalu^2^, Kristiina Rull^1,2^, Maris Laan^1*^

Affiliations:

^1^ Human Molecular Genetics group, Institute of Molecular and Cell Biology, University of Tartu,

Riia 23, 51010 Tartu, Estonia

^2^ Women’s Clinic of Tartu University Hospital, L. Puusepa 8, 51014 Tartu, Estonia

**Corresponding author:**

*Maris Laan, PhD, Human Molecular Genetics group, Institute of Molecular and Cell Biology, University of Tartu; Riia St. 23,Tartu 51010, Estonia. Tel: +372-7375008; Fax: +372-7420286; e-mail: maris.laan@ut.ee

**Supplemental Data files:**

1. **MANNIK et al. Suppl. text :**

- **Supplemental Materials and Methods**
- **Supplemental Figure legends**

1. **MANNIK et al. Suppl. Fig S1-Fig S2 – Supplemental figure 1 and figure 2**

**Supplemental Materials and Methods**

***RT-PCR reaction conditions***

Each PCR reaction was performed in a total volume of 25 μl containing 2.5 μl 10xPCR reaction buffer B (Solis BioDyne, Estonia), 2 mM MgCl_2_, 0.2 mM dNTP mix (Solis BioDyne), 0.5 units HOT FIREPol® DNA Polymerase (Solis BioDyne), cDNA (0.5 μl), and 400 nmol/μl of forward and reverse primers. For consistency, all PCR reagents (except primers) and cDNA were combined in one tube for all gene specific and reference gene reactions, mixed thoroughly, distributed equally between PCR tubes and finally gene-specific primers were added.

Amplification was attained by GeneAmp PCR System 2700 (Applied Biosystems Inc., USA) under the following conditions: 95°C for 15 min to denature, then 10 cycles of 95°C for 20s to denature, and from 67 to 56°C for 30s (‘touch-down’) to anneal, and 72°C for 1 min to extend, followed by 15 cycles of 95°C for 20s, 56°C for 30s and 72°C for 1 min (for *CSH1/CSH2* genes). In case of *GAPDH* and *GH2* transcripts 20 cycles and in case of *GH2-2* transcript 22 cycles were performed under conditions described above. In the final the extension time was increased to 10 min. The number of cycles needed for amplification for each assay was optimized to ensure that PCR remained in the logarithmic phase of amplification.

**Supplemental Figure legends**

**Supplemental figure 1**

Comparison of relative expression levels of major alternatively spliced mRNA transcripts of *GH2* (A-C), *CSH1* (D-E), and *CSH2* (F-G) in placentas from uncomplicated pregnancies grouped by newborn gender. Relative expression of each transcript is given as ratio to the reference gene *GAPDH*. The boxes represent the 25th and 75th percentiles. The median is denoted as the line that bisects the boxes. The whiskers are lines extending from each end of the box covering the extent of the data on 1.5 X interquartile range. Circles represent the outlier values. Statistical differences between study groups were assessed by Mann-Whitney U test and all comparisons resulted in *P*>0.1.

**Supplemental figure 2**

Comparison of relative expression levels of *GH2-1* (A), *GH2-3* (B), *CSH1-1* (C), *CSH2-1* (D) and *CSH2-2* (E) in placentas from uncomplicated pregnancies (Controls, n=17) and pre-eclampsia (PE) cases grouped by the fetal growth - PE with the birth of appropriate-for-gestational age newborn (PE-AGA, n=9) and PE with the birth of small-for-gestational age newborn (PE-SGA, n=8). Relative expression of each transcript is given as ratio to the reference gene *GAPDH*. The boxes represent the 25th and 75th percentiles. The median is denoted as the line that bisects the boxes. The whiskers are lines extending from each end of the box covering the extent of the data on 1.5 X interquartile range. Circles represent the outlier values. Plotted values are presented without covariate adjustment. Statistical differences between study groups were assessed by ANCOVA using Bonferroni correction. Statistical tests were adjusted for gestational age. No statistical difference (*P*>0.1) between the groups was detected.
